# Involvement of neuronal and muscular Trk-fused gene (TFG) defects in the development of neurodegenerative diseases

**DOI:** 10.1038/s41598-022-05884-7

**Published:** 2022-02-04

**Authors:** Takeshi Yamamotoya, Shun Hasei, Yasuyuki Akasaka, Yukino Ohata, Yusuke Nakatsu, Machi Kanna, Midori Fujishiro, Hideyuki Sakoda, Hiraku Ono, Akifumi Kushiyama, Hidemi Misawa, Tomoichiro Asano

**Affiliations:** 1 Department of Biomedical Chemistry, Graduate School of Biomedical and Health Sciences, Hiroshima University, 1-2-3 Kasumi, Minami-ku, Hiroshima City, Hiroshima 734-8551 Japan; 2 Division of Diabetes and Metabolic Diseases, Nihon University School of Medicine, 30-1 Oyaguchikamicho, Itabashi, Tokyo 173-8610 Japan; 3 Division of Neurology, Respirology, Endocrinology and Metabolism, Department of Internal Medicine, Faculty of Medicine, University of Miyazaki, 5200 Kihara, Kiyotake, Miyazaki, 889-1692 Japan; 4 Department of Clinical Cell Biology, Graduate School of Medicine, Chiba University, 1-8-1 Inohana, Chuo-ku, Chiba City, Chiba 260-8670 Japan; 5 Department of Pharmacotherapy, Meiji Pharmaceutical University, 2-522-1 Noshio, Kiyose City, Tokyo 204-8588 Japan; 6 Division of Pharmacology, Faculty of Pharmacy, Keio University, 1-5-30, Shibakoen, Minato-ku, Tokyo, 105-8512 Japan

**Keywords:** Neuroscience, Neurology

## Abstract

Trk-fused gene (TFG) mutations have been identified in patients with several neurodegenerative diseases. In this study, we attempted to clarify the effects of TFG deletions in motor neurons and in muscle fibers, using tissue-specific TFG knockout (vMNTFG KO and MUSTFG KO) mice. vMNTFG KO, generated by crossing TFG floxed with VAChT-Cre, showed deterioration of motor function and muscle atrophy especially in slow-twitch soleus muscle, in line with the predominant Cre expression in slow-twitch fatigue-resistant (S) and fast-twitch fatigue-resistant (FR) motor neurons. Consistently, denervation of the neuromuscular junction (NMJ) was apparent in the soleus, but not in the extensor digitorum longus, muscle. Muscle TFG expressions were significantly downregulated in vMNTFG KO, presumably due to decreased muscle IGF-1 concentrations. However, interestingly, MUSTFG KO mice showed no apparent impairment of muscle movements, though a denervation marker, AChRγ, was elevated and Agrin-induced AChR clustering in C2C12 myotubes was inhibited. Our results clarify that loss of motor neuron TFG is sufficient for the occurrence of NMJ degeneration and muscle atrophy, though lack of muscle TFG may exert an additional effect. Reduced muscle TFG, also observed in aged mice, might be involved in age-related NMJ degeneration, and this issue merits further study.

## Introduction

The Trk-fused gene (TFG) was originally identified as part of an oncogene termed TRK-T3, in which the N-terminal half of TFG was fused with neurotrophic receptor tyrosine kinase 1 (NTRK1, also known as TrkA) in 1995^1^. Subsequent studies revealed TFG to be a fusion partner of other cancer related genes such as anaplastic lymphoma kinase (ALK) in anaplastic large cell lymphoma^2^, RARA in acute promyelocytic leukemia^3^ and RET in spindle cell tumor^4^ and papillary thyroid cancer^5^. As implied by its high sequence homology among species^6^ and its ubiquitous expression, TFG itself plays physiologically essential roles in cell biology. In 2011, TFG was reported to be essential for forward protein transport from the endoplasmic reticulum (ER) to ER-Golgi intermediate compartments (ERGIC) via COPII vesicles^7^. TFG octamers form cup-like structures, localize between ER and ERGIC, and upon depletion of TFG, the tight association between ER and ERGIC was lost and COPII vesicles were scattered throughout the cytoplasmic compartments^8^, since TFG competitively binds to inner coat protein Sec23 and facilitates outer coat disassembly of COPII vesicles, a necessary step for subsequent tethering and fusion with ERGIC^9^.

Independently of these findings, TFG was recently revealed to be a causal gene of several neurodegenerative diseases, including hereditary motor and sensory neuropathy with proximal dominant involvement (HMSN-P)^10^, the axonal type of Charcot-Marie-Tooth disease (CMT2)^11^ and hereditary spastic paraplegia (HSP)^12^. HMSN-P is an adult-onset autosomal-dominant neurodegenerative disease which is characterized by proximal dominant neurogenic atrophy, distal sensory loss and fasciculations^10,13^. The most widely known mutation responsible for this disease is the heterozygous p.Pro285Leu mutation in the P/Q rich domain of TFG, which causes cytoplasmic aggregation of TAR DNA-binding protein 43 kDa (TDP-43)^10^ and ubiquitin proteasome system impairment^14,15^, both of which are attributed to the toxicity of the mutant form of TFG. Heterozygous p.Gly269Val mutation of TFG reportedly causes CMT2^11^. The p.Gly269Val mutants were found to be prone to form insoluble aggregates, also leading to partial depletion of functional wild-type TFG and thereby compromising protein secretion^11^. While HMSN-P and CMT2 require mutation of a single allele, biallelic TFG mutations, such as p.Arg106Cys, underlie the pathogenesis of HSP and several mechanisms, including mitochondrial fragmentation^16^, defects in protein transport and axon bundling^17^, have been proposed to date. Although studies using homozygous mutant p.Arg106Cys TFG cells are of great value not only for elucidating the pathogenesis of HSP but also for studying the consequences of the loss of functional TFG, the knowledge obtained from TFG-knockout (KO) mouse models is necessary for understanding the physiological functions of TFG and neurodegenerative disorders caused by mutations affecting this gene.

In a previous study^18^, our group generated TFG floxed mice and reported the importance of TFG in the regulation of pancreatic β cell mass and functions. By crossing with VAChT-Cre.Early mice^19,20^, we generated postnatal motor neuron-specific TFG KO mice and report herein that deletion of TFG in the motor neuron results in denervation of neuromuscular junctions (NMJs) as well as muscle atrophy. Furthermore, we propose a possible contribution of muscle TFG, downregulated upon denervation or with aging, to the physiological maintenance of NMJs.

## Materials and methods

### Mice

Motor neuron-specific TFG KO mice (MNTFG KO) were generated by crossing TFG floxed mice^18^ with VAChT-Cre.Early mice^19,20^. Since VAChT-Cre mice were recently demonstrated to express Cre recombinase specifically in slow-twitch fatigue-resistant (S) and fast-twitch fatigue-resistant (FR) motor neurons^20^, but in neither fast-twitch fatigable (FF) nor γ-type motor neurons, we use the designation vMNTFG KO to describe TFG f/f; VAChT-Cre.Early mice herein. Muscle-specific TFG KO mice (MUSTFG KO) were generated by crossing TFG floxed mice with transgenic mice expressing Cre under a muscle-specific alpha-actin promoter provided by RIKEN BRC (MuCre-A mice, RBRC01386)^21^. All mice used in this study had the C57BL/6 genetic background. Six- to eight-month-old male mice were used for experiments unless otherwise noted. All animals were handled in accordance with ARRIVE guidelines and the Guidelines for the Care and Use of Experimental Animals published by Hiroshima University and all protocols were approved by the Institutional Review Board of Hiroshima University.

### Motor function tests

For the treadmill tests (Muromachi Kikai, Tokyo, Japan), mice were forced to start running at 10 m/min and continue at this pace for 5 min. The speed was then increased by 5 m/min every 5 min until it reached 25 m/min. The test was discontinued either when the mice were touching the electrode at the rear of the treadmill for more than 5 s or they had run for 90 min in total. In the hanging-wire test, mice were placed on a grid, which was then inverted, and the latency to fall was measured twice, with the maximum value being recorded.

### Western blotting

50 mg of muscle tissues were homogenized with 1 ml of lysis buffer containing 50 mM Tris–HCl (pH 7.4), 150 mM NaCl, 1 mM ethylenediamine tetraacetic acid **(**EDTA), 1% Triton X-100, 1 mM NaF, 1 mM Na_3_VO_4_, and 1 mM phenylmethylsulfornyl fluoride (PMSF). After incubation on ice for 30 min, samples were centrifuged at 15,000 rpm for 10 min and subsequently for 30 min at 4 °C. After adjusting the protein concentrations, the supernatants were mixed and boiled with sample buffer containing sodium dodecyl sulfate (SDS) and 2-mercaptoethanol. Samples were electrophoresed with SDS–polyacrylamide gel, transferred to PVDF (polyvinylidene difluoride) membranes and subjected to immunoblotting using SuperSignal West Pico PLUS Chemiluminescent Substrate (Thermo Scientific, Waltham, MA, USA). The antibodies used were anti-TFG (1:10,000, ab156866, Abcam) and anti-actin (1:2000, sc-47778, Santa Cruz).

### Quantitative real-time PCR

Total RNA was extracted using Sepasol-RNA I Super G (Nacalai Tesque, Kyoto, Japan) and first-strand cDNA was obtained using a Verso cDNA Synthesis Kit (Thermo Scientific), according to the manufacturers’ instructions. Real-time PCR was performed using the CFX96 real-time PCR system (Bio-Rad, Hercules, CA, USA) with Brilliant III Ultra-Fast SYBR Green QPCR Master Mix (Agilent, Santa Clara, CA, USA). *Gapdh* was used as the reference gene. The primers used are listed in Supplementary Table S1.

### Immunohistochemistry

The mice were anesthetized, perfused with phosphate buffered saline (PBS) and subsequently with 4% formaldehyde in PBS. Tissues were excised and immediately fixed with 4% formaldehyde in PBS overnight and embedded in paraffin. Sections were cut, deparaffinized, permeabilized with 0.1% Triton and heated in 10 mM citrate buffer (pH 6.0) using a microwave for antigen retrieval. Diaminobenzidine (DAB) staining was performed using a VECTASTAIN Elite ABC Kit (Vector Labs, Burlingame, CA, USA) and ImmPACT DAB (Vector Labs) according to the manufacturer’s instructions. For fluorescent immunostaining, sections were blocked with 3% goat serum in PBS for 1 h, and then incubated with primary antibodies diluted in Can Get Signal Immunostain Solution (Toyobo, Osaka, Japan) overnight at 4 °C. Sections were then incubated with secondary antibodies conjugated with fluorescent dye for 1 h, and then mounted using a Vector TrueVIEW Autofluorescence Quenching Kit (Vector). The primary antibodies used were anti-myosin (skeletal, slow) (1:400, M8421, SIGMA), anti-ChAT (1:5000 for DAB staining, provided by Dr. Hidemi Misawa^22^), anti-ChAT (1:1000 for fluorescent immunostaining, MA5-31383, Invitrogen), anti-TFG (1:100, ab156866, Abcam) and anti-OPN (1:100, sc-21742, Santa Cruz).

### Whole-mount staining

Mice were fixed with 4% formaldehyde perfusion as described above. Soleus and extensor digitorum longus (EDL) muscles were excised, longitudinally teased apart into several pieces and then blocked for 1 h in PBS containing 3% bovine serum albumin (BSA), 5% goat serum and 0.5% Triton. Then, the samples were incubated overnight at 4 °C with anti-synaptophysin antibody (1:500, ab32127, Abcam) in antibody dilution buffer (PBS with 3% BSA, 5% goat serum, 0.1% Triton). After being washed three times with PBS, the samples were incubated for 2 h with Cy3-conjugated secondary antibody (1:400) and Alexa Fluor 488-conjugated α-bungarotoxin (1:200, B13422, Invitrogen) in antibody dilution buffer. After being washed three times with PBS, the samples were mounted as described above.

### Measurement of serum and muscle IGF-I concentrations

Serum IGF-I concentrations were measured using a Mouse/Rat IGF-I Quantikine ELISA Kit (R&D Systems, Minneapolis, MN, USA) according to the manufacturer’s instructions. For the measurement of muscle IGF-I concentrations, 50 mg of gastrocnemius muscle were homogenized with 1 ml of lysis buffer containing 50 mM Tris–HCl (pH 7.4), 150 mM NaCl, 1 mM EDTA, 1% Triton X-100, 1 mM NaF, 1 mM Na_3_VO_4_, and 1 mM PMSF. After incubation on ice for 30 min, the samples were centrifuged at 15,000 rpm for 10 min and subsequently for 30 min at 4 °C. IGF-I concentrations of the supernatants were measured and the results were adjusted by the protein concentrations of the supernatants.

### Agrin-induced AChR clustering

C2C12 cells were cultured with Dulbecco’s Modified Eagle Medium (DMEM) containing 10% fetal bovine serum and were differentiated into myotubes by switching the medium to DMEM containing 2% horse serum (day 0). At day 2, cells were transfected with siRNA using Lipofectamine RNAiMAX Transfection Reagent (Thermo Scientific). At day 5, recombinant rat Agrin (R&D Systems) was added at a concentration of 100 ng/ml. After being incubated overnight and fixed with 2% formaldehyde in PBS, AChR clustering was detected by staining with Alexa 488 conjugated α-bungarotoxin (1:200, Invitrogen) for 2 h at room temperature.

### Statistical analysis

Statistical analyses were performed using Microsoft Excel or EZR (Saitama Medical Center, Jichi Medical University, Saitama, Japan)^23^. Values are presented as means ± SE. We used student’s unpaired t-test for comparing two groups, and one-way ANOVA followed by the post-hoc Dunnett’s test for multiple comparisons. We considered *P* < 0.05 to indicate a statistically significant difference.

## Results

### vMNTFG KO mice display deterioration of motor function

In order to clarify the consequences of TFG KO in motor neurons, we first attempted to generate neuron-specific TFG KO mice by crossing TFG floxed mice with Nestin-Cre transgenic mice. However, mating TFG f/+; Nestin-Cre with TFG f/f did not result in the birth of any TFG f/f; Nestin-Cre mice, suggesting neuronal TFG to be essential for embryonic development. To circumvent the lethality of deleting TFG during embryonic development, we utilized VAChT-Cre.Early mice, in which Cre expression is driven by the 11.3 kb human vesicular acetylcholine transporter (VAChT) promoter and starts approximately 7 days after birth^19,20^. In VAChT-Cre mice, Cre expression is restricted to a limited fraction of postnatal somatomotor neurons^19^, which were recently revealed to mainly be slow-twitch fatigue-resistant (S) and fast-twitch fatigue-resistant (FR) motor neurons^20^. Hence, TFG f/f; VAChT-Cre.Early mice lack TFG predominantly in these subsets of motor neurons, rather than in motor neurons in general. Therefore, we use the designation vMNTFG KO to represent TFG f/f; VAChT-Cre.Early mice herein.

**Figure 1:**
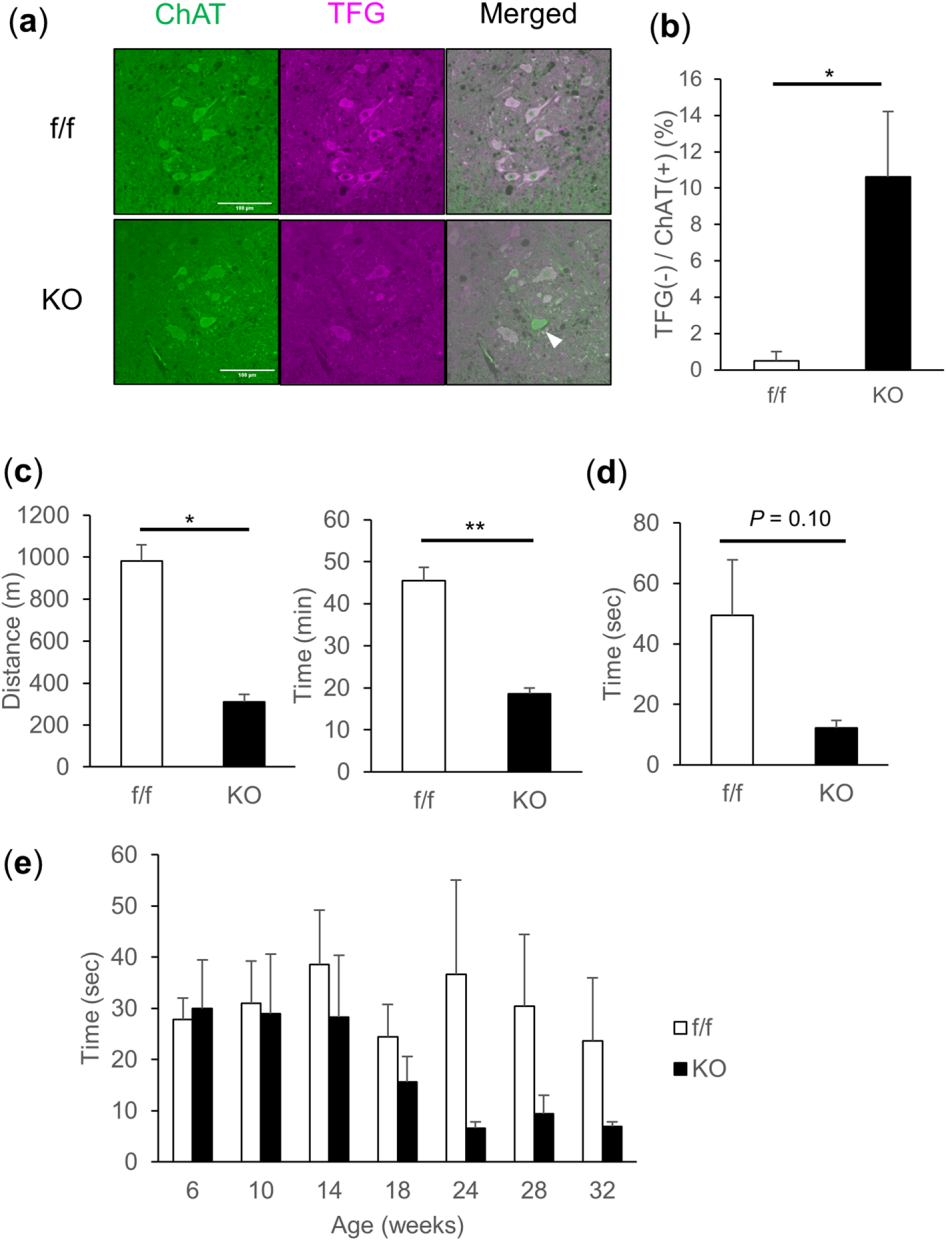
vMNTFG KO mice display reduced motor function (muscle weakness). (**a**) Fluorescent immunostaining against ChAT and TFG in lumbar spinal cord specimens from 6-month-old mice (scale bar: 100 µm). ChAT-positive but TFG-negative motor neuron in vMNTFG KO is indicated by arrowhead. (**b**) Percentage of TFG-negative neurons among ChAT-positive motor neurons. ChAT-positive motor neurons number 213 and 177 from f/f (n = 6) and KO (n = 5), respectively. (**c**) Treadmill test results of 4-month-old male mice (n = 4). Distance (left panel) and time (right panel) that mice ran are shown. (**d**) Latency to fall in hanging-wire test results of 4-month-old male mice (n = 4–6). (**e**) Chronological changes in performance of the hanging-wire test (n = 8–9). (*P < 0.05, **P < 0.01).

Immunostaining of lumbar spinal sections from vMNTFG KO using antibodies against TFG, together with choline acetyltransferase (ChAT), which is expressed in motor neurons in general, revealed TFG-negative motor neurons (Fig. 1a). TFG-negative motor neurons accounted for approximately 10% of ChAT-positive motor neurons in vMNTFG KO (Fig. 1b) and were predominantly osteopontin (OPN)-high neurons (Suppl Fig. S1a), with OPN being a recently reported marker for S and FR motor neurons^24^. However, there was no discernable difference in the protein expression levels of TFG in whole cell lysates from the brain or spinal cord between the genotypes similarly as in those from other tissues such as the liver, heart and lungs (Suppl Fig. S1b-f).

Initially, we examined motor functions in vMNTFG KO using treadmill and hanging-wire tests. The treadmill test was performed at 4 months of age and revealed significantly lower endurance in vMNTFG KO (Fig. 1c). Latency to fall in the hanging-wire test also showed a tendency for vMNTFG KO to have less strength than normal mice (Fig. 1d), and this had become quite obvious by the age of 6 months (Fig. 1e). Therefore, we performed all subsequent analyses using 6- to 8-month-old mice.

### vMNTFG KO mice manifest slow muscle atrophy and decreased slow-twitch fibers

**Figure 2:**
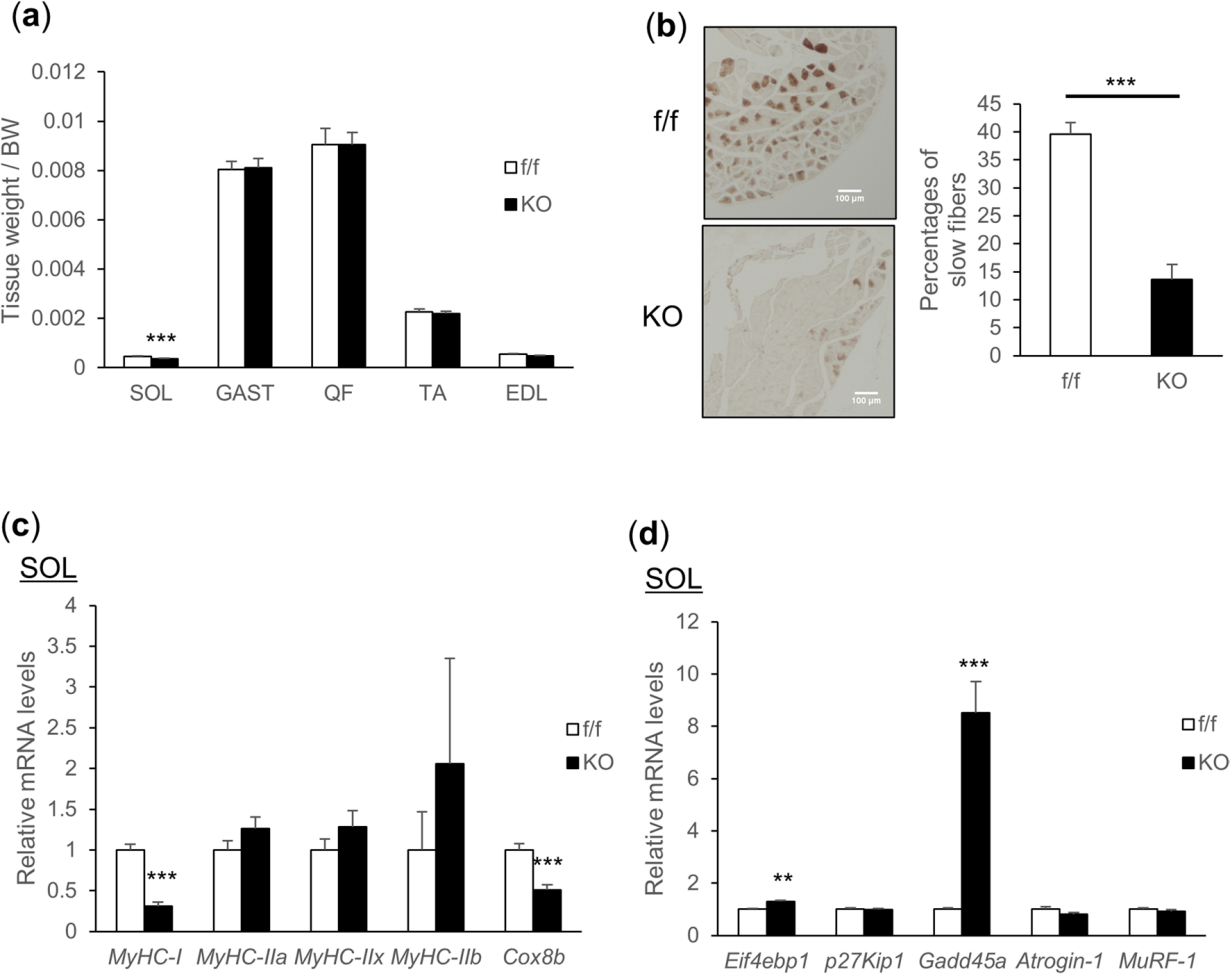
vMNTFG KO mice manifest muscle atrophy especially in slow-twitch fibers. (**a**) Muscle mass (bilateral) adjusted by body weight (7-month-old, n = 7–8). SOL: soleus, GAST: gastrocnemius, QF: quadriceps femoris, TA: tibialis anterior, EDL: extensor digitorum longus. (**b**) Representative immunostaining of SOL against myosin heavy chain (MyHC) type 1 (left panel) (scale bar: 100 µm) and the percentage of DAB-positive slow-fibers (right panel) (6-month-old mice, n = 5). (**c**) mRNA levels of each myosin-heavy chain (MHC) isoform and the mitochondrial gene Cox8b in SOL (7-month-old mice, n = 7–8). (**d**) mRNA levels of genes related to muscle atrophy in SOL (7-month-old mice, n = 7–8). (**P < 0.01, ***P < 0.001).

While there was no difference in body weight (Suppl Fig. S2a), soleus muscle mass was significantly smaller in vMNTFG KO than in the controls (Fig. 2a). Immunostaining of soleus muscle using antibodies against myosin heavy chain (MyHC) type 1 revealed the percentage of slow fibers to be significantly decreased in soleus muscles from vMNTFG KO mice (Fig. 2b, Suppl Fig. S2b). Consistently, mRNA levels of MyHC type 1 were specifically downregulated in soleus from vMNTFG KO (Fig. 2c). mRNA levels of the mitochondrial marker cytochrome c oxidase subunit 8B (Cox8b) were also significantly reduced in soleus muscles (Fig. 2c), consistent with the decrease in slow-twitch fibers rich in mitochondria. Furthermore, expressions of several atrogenes, such as the growth arrest and DNA damage-inducible protein GADD45 alpha (Gadd45a), were significantly upregulated in soleus muscles (Fig. 2d). Although less striking, downregulation of MyHC type 1 and upregulation of atrogenes were also observed in gastrocnemius muscle comprised of both slow- and fast-twitch fibers (Suppl Fig. S2c), but not in the fast-twitch tibialis anterior muscle (Suppl Fig. S2d). These data indicate that vMNTFG KO manifest slow muscle atrophy and a decrease in slow-twitch fibers, presumably reflecting the Cre expression preference in VAChT-Cre.Early mice.

### vMNTFG KO show denervation of neuromuscular junctions (NMJs) predominantly in slow fibers

Choline acetyltransferase (ChAT) staining of the lumbar spinal cord showed no apparent decrease in the number of ChAT-positive neurons in the ventral horns of lumbar spinal cord from vMNTFG KO (Suppl Fig. S3a). Furthermore, toluidine blue staining (Suppl Fig. S3b) and electron microscopic observation (Suppl Fig. S3c) of sciatic nerves revealed no apparent differences between vMNTFG KO and the controls.

**Figure 3:**
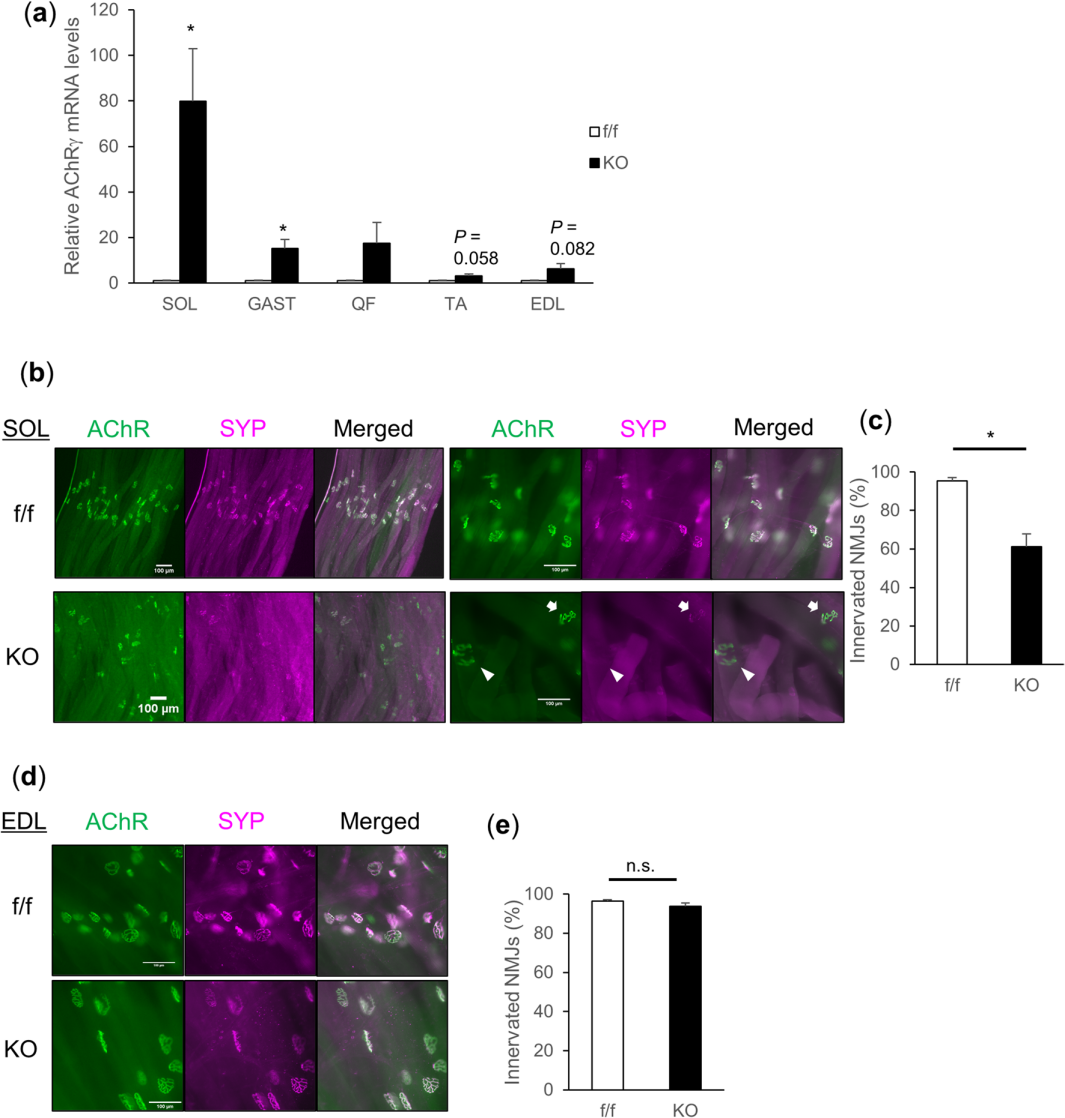
TFG deletion in motor neuron results in denervation of neuromuscular junctions. (**a**) Relative *AChR*γ mRNA levels in SOL, GAST, QF, TA and EDL muscles (6-month-old mice, n = 4–5). (**b**, **d**) Whole-mount staining of SOL (**b**) and EDL (**d**) muscles (scale bar: 100 µm) (7-month-old mice). Green: AChR stained by α-bungarotoxin. Magenta: Synaptophysin as a pre-synaptic marker. White arrowhead indicates completely denervated NMJ and white arrow indicates partially denervated NMJ. (**c**, **e**) Percentage of innervated neuromuscular junctions in SOL (**c**) and EDL (**e**) muscles (7-month-old mice, n = 4–5). Both completely and partially innervated NMJs were defined as being innervated. (*P < 0.05).

Intriguingly, mRNA levels of acetylcholine receptor gamma subunit (AChRγ), which is a fetal subunit of AChR and is also induced in denervated muscles^25^, were dramatically upregulated in muscles, especially the soleus, from vMNTFG KO (Fig. 3a). Whole-mount staining of soleus muscle tissue using α-bungarotoxin, which binds to AChRs, and antibodies against synaptophysin, a presynaptic marker, revealed apparent denervation of motor endplates in vMNTFG KO (Fig. 3b,c). Of note, while AChRγ mRNA showed a trend toward upregulation (Fig. 3a), histological NMJ denervation was not observed in fast-twitch EDL muscles (Fig. 3d,e), suggesting that denervation of NMJs occurs predominantly in slow fibers as a consequence of TFG deletion in the innervating nerves.

### TFG is downregulated in muscles from vMNTFG KO, possibly via decreased muscle IGF-1 concentrations

**Figure 4:**
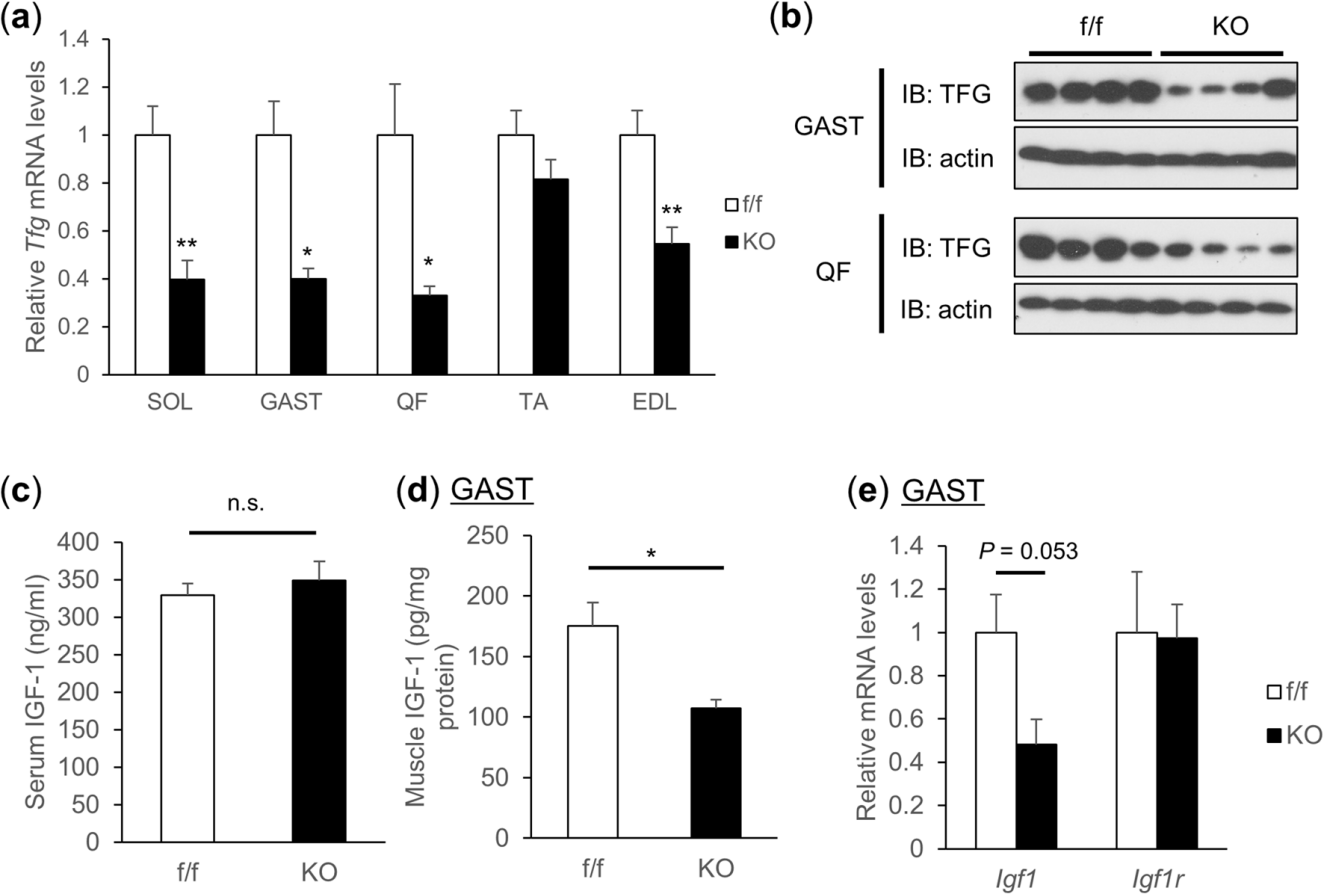
TFG is downregulated in muscles from vMNTFG KO mice. (**a**) Relative *Tfg* mRNA levels in each muscle (6-month-old, n = 5). (**b**) Western blotting of gastrocnemius (GAST) and quadriceps femoris (QF) muscle samples (6-month-old mice). (**c**) Serum IGF-1 concentrations (8-month-old mice, n = 5). (**d**) IGF-1 concentrations in GAST homogenates (8-month-old mice, n = 5, adjusted by the protein concentration). (**e**) Relative *Igf1* and *Igf1r* mRNA levels in GAST (6-month-old mice, n = 4–5). (*P < 0.05, **P < 0.01).

Interestingly, we found that TFG expressions were downregulated in muscles from vMNTFG KO as compared to controls, in terms of both mRNA (Fig. 4a) and protein abundance (Fig. 4b). In reference to a recent study which found TFG expression levels to be regulated by IGF-1 in sebocytes^26^, we focused on the serum and muscle IGF-1 levels of vMNTFG KO. Whereas there was no difference in serum IGF-1 levels (Fig. 4c), IGF-1 concentrations in gastrocnemius muscle were significantly lower in vMNTFG KO than in the controls (Fig. 4d), which might explain the lower TFG expression levels in muscles from vMNTFG KO. Consistently, IGF-1 mRNA levels showed a trend toward lower expressions in gastrocnemius muscle from vMNTFG KO (Fig. 4e), with no effect on IGF-1R mRNA levels.

### Muscle TFG is not essential for motor functions but appears to be important in NMJ maintenance

To determine the contributions of the documented downregulation of TFG in muscle to the deterioration of motor functions and muscle atrophy observed in vMNTFG KO, we generated muscle-specific TFG KO mice (MUSTFG KO) and analyzed their phenotypes. In contrast to vMNTFG KO, MUSTFG KO showed no impairment of motor functions at least at 3 months of age (Suppl Fig. S4a, b).

When the mice were analyzed at 6 to 7 months of age, body weight was slightly higher (Suppl Fig. S4c) and there was no apparent muscle atrophy in MUSTFG KO as compared to the controls (Suppl Fig. S4d). Gastrocnemius IGF-1 concentrations were similar in the two groups (Suppl Fig. S4e), indicating that the decreased muscle IGF-1 concentrations observed in vMNTFG KO did not originate from decreased muscle TFG.

**Figure 5:**
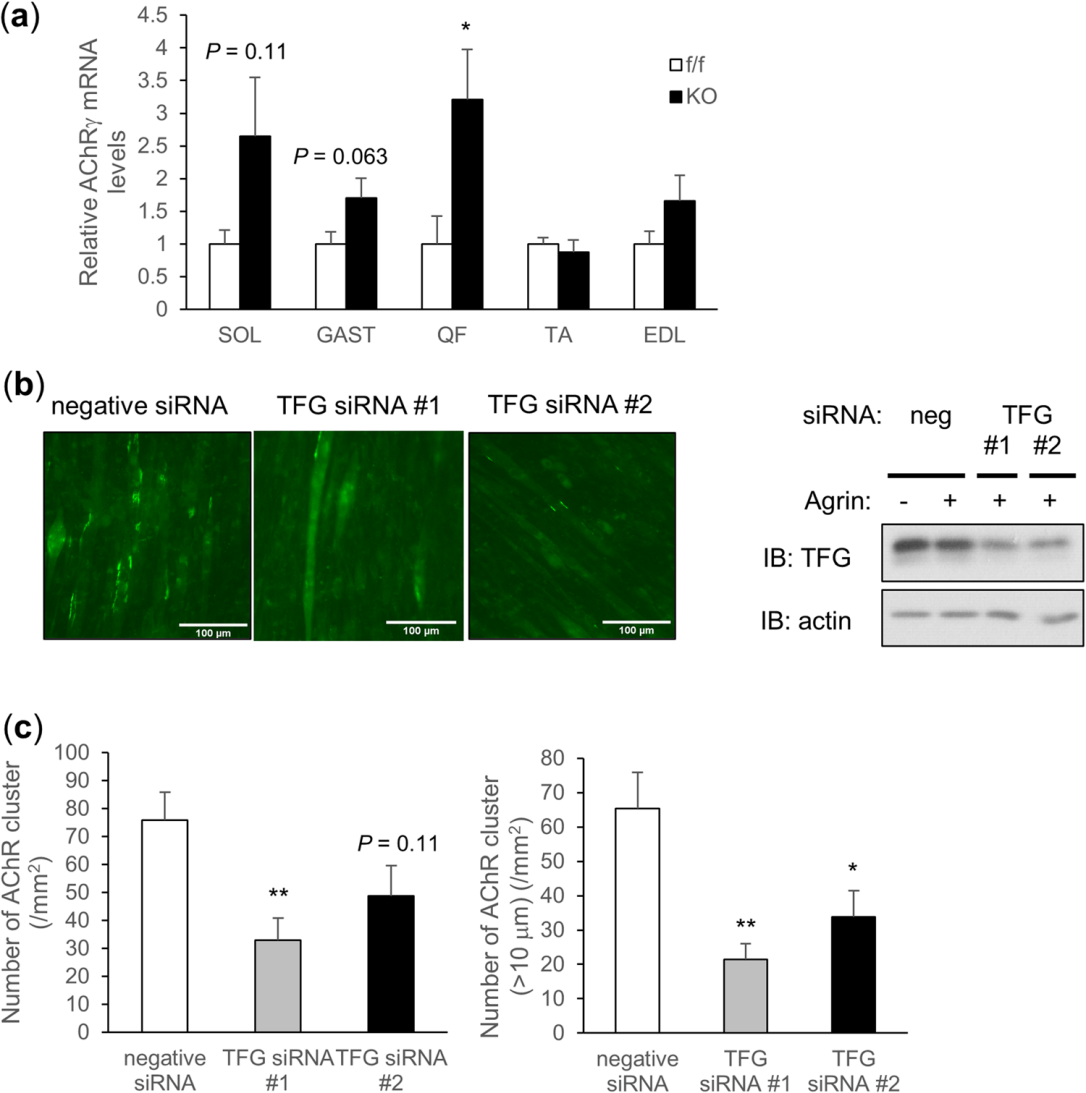
Muscle TFG might be important for neuromuscular junction (NMJ) maintenance. (**a**) Relative *AChR*γ mRNA levels in each muscle from TFG floxed (f/f) or MUSTFG KO (KO) mice (6- to 7-month-old mice, n = 10–11). (**b**) Agrin-induced AChR clustering in C2C12 myotubes (left panel) (scale bar: 100 µm) and protein abundance of TFG in Western blot (right panel). (**c**) The number of AChR clusters per area (left panel) and the number of AChR clusters larger than 10 µm in diameter (right panel). (*P < 0.05, **P < 0.01).

Surprisingly, however, mRNA levels of a denervation marker, AChRγ, though not as markedly as in vMNTFG KO, were upregulated in several muscles from MUSTFG KO as compared to the controls and the increase was statistically significant in the quadriceps femoris muscle (Fig. 5a). However, this upregulation of AChRγ mRNA was not as marked as in vMNTFG KO (Fig. 3a) and whole-mount NMJ staining of soleus muscles from MUSTFG KO (in which AChRγ mRNA levels are 2.6-fold higher than those in f/f) did not reveal histologically apparent denervation (Suppl. Fig. S4f).

Agrin secreted from motor neurons is essential not only for NMJ formation but also for its maintenance in adulthood^27–30^. Interestingly, agrin-induced AChR clustering in C2C12 myotubes was significantly impaired when TFG was knocked down by siRNA transfection, which was especially prominent when AChR clusters larger than 10 µm were assessed (Fig. 5b,c).

**Figure 6:**
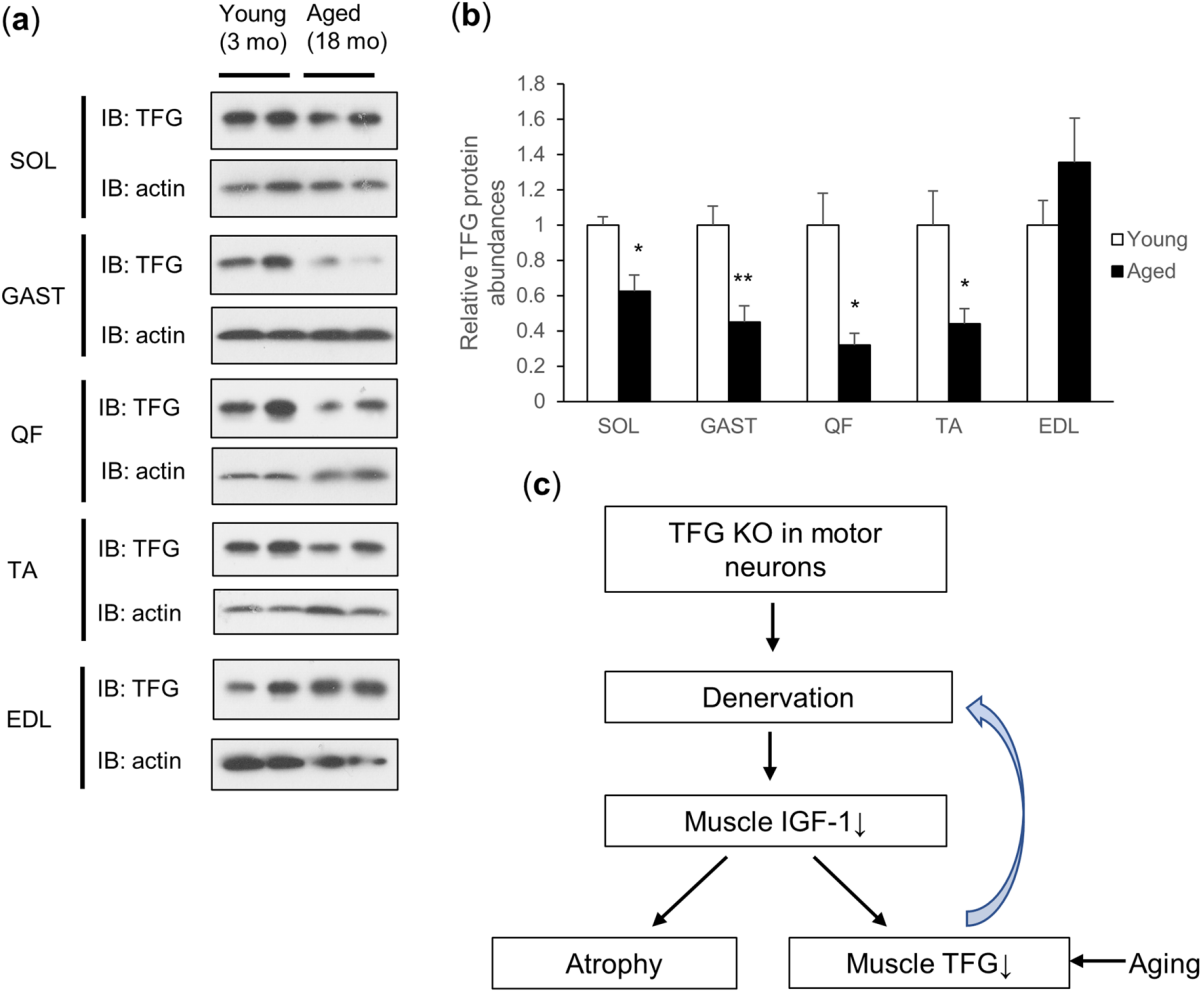
Muscle TFG decreases with aging. (**a**) Western blotting of SOL, GAST, QF, TA and EDL muscle samples from 3-month-old (young) and 18-month-old (aged) TFG f/f male mice. (**b**) Quantification of TFG protein abundances (adjusted by actin) (n = 5). (**c**) Summary of this study. (*P < 0.05, **P < 0.01).

Finally, we assessed the expression levels of muscle TFG from young (3-month-old) and aged (18-month-old) mice, which revealed a marked decline of TFG expression with aging in most of the muscles investigated (Fig. 6a,b). These data raise the possibility that muscle TFG also plays important roles in the maintenance of NMJs and that the decreased muscle TFG observed in aged mice might contribute to age-related NMJ degeneration^28^.

## Discussion

In this study, we generated vMNTFG KO mice, in which deletion of the TFG gene is intended to occur postnatally and predominantly in slow-twitch fatigue-resistant (S) and fast-twitch fatigue-resistant (FR) motor neurons^19,20^. As expected, vMNTFG KO mice showed marked weakness, i.e., muscle function was clearly diminished, based on the treadmill and hanging-wire test results performed at 4 months of age. In soleus and gastrocnemius muscles, assumed to be controlled by neurons expressing Cre, NMJ denervation was observed, which resulted in significant muscle atrophy in the soleus. While previous reports highlighted the toxic effects of the mutant TFG as causes of the pathogenesis of HMSN-P^10,14,15^, our findings that at least some of the motor symptoms observed in HMSN-P were recapitulated in vMNTFG KO (i.e., muscle atrophy, muscle weakness) raise the possibility that the pathogenesis of HMSN-P might involve both the loss of functional TFG and direct toxic effects of the mutant TFG, rather than necessarily being attributable entirely to the latter.

An important question is whether postnatal TFG deletion in motor neurons is detrimental enough to cause motor neuron loss. ChAT staining of lumbar spinal sections revealed no apparent motor neuron loss in vMNTFG KO (Suppl Fig. S3a). However, the percentage of TFG-negative ChAT-positive motor neurons in vMNTFG KO was just above 10% (Fig. 1b), i.e., apparently lower than expected. Given that S or FR motor neurons account for 20% of motor neurons^24^ and that the percentage of Cre-expressing S or FR motor neurons in VAChT-Cre.Early was 78.0% and that this accounts for 70–80% of Cre expressing cells in spinal cord sections^20^, the percentage of TFG-negative motor neurons may well approach 20% if all of the TFG-negative motor neurons remained viable. This suggests roughly half of TFG-deleted motor neurons to be subjected to cell death and to be absent when assessed at 6 months of age. To further verify this observation, we performed ChAT/OPN staining of lumbar spinal sections to determine whether the numbers of S or FR motor neurons were specifically reduced in vMNTFG KO. Although not statistically significant, the proportion of OPN-high motor neurons was apparently lower in vMNTFG KO than in the controls (Suppl Fig. S3d, e), which supports the hypothesis that at least some of the TFG-deleted motor neurons had been lost by 6 months of age. This is in contrast to our previous study of pancreatic β-cells^18^, in which no apparent increase in cell death was observed upon TFG deletion. Given that all human diseases caused by TFG mutations, reported to date, are neurodegenerative disorders, it is understandable that TFG is indispensable especially for neurons and TFG deletion in neurons increases susceptibility to cell death.

Immunostaining of MyHC type 1 revealed the proportion of type 1 fibers in soleus muscles to be decreased by approximately two-thirds in vMNTFG KO as compared to the controls (Fig. 2b), which corresponded well with the results of *MyHC-I* mRNA quantification (Fig. 2c). Interestingly, type 1 fibers were scattered throughout the sections from control mice, whereas they were localized to a part of the sections from at least some of the KO mice (typically observed in KO #3, #5) (Suppl. Fig. S2b). We surmise that upon motor neuron denervation and/or subsequent cell death, motor units innervated by S motor neurons were reinnervated by sprouting from neighboring FF or possibly FR motor neurons, which results in a fiber type switch^31–33^. The significant upregulation of *MyHC-IIx* mRNA levels observed in gastrocnemius muscles might support this hypothesis (Suppl Fig. S2c).

An important finding in this study is that muscle TFG expressions in vMNTFG KO mice were also downregulated in most of the muscles investigated, which might be explained by reduced muscle IGF-1 concentrations. Apart from systemic IGF-1 which is produced in the liver, IGF-1 locally produced in muscles has attracted considerable research attention in terms of the therapeutic potentials for neurodegenerative diseases such as ALS^34,35^. Since muscle contraction (produced by either an electrical stimulus or exercise) induces IGF-1 expression^36^, the lower muscle IGF-1 concentration in vMNTFG KO might reflect less frequent muscle contraction due to denervation and thereby the lower locomotor activity observed in affected individuals (Fig. 1b), given that TFG expressions were also downregulated in fast-twitch muscles such as EDL (Fig. 4a). However, further studies are needed to clarify the underlying mechanisms.

We speculated that the muscle TFG reduction observed in vMNTFG KO might also contribute to motor dysfunction, given the fundamental role of TFG in cell function and its abundant expression. Therefore, in addition to vMNTFG KO, we also generated MUSTFG KO mice lacking muscle TFG and endeavored to ascertain whether reduced muscle TFG expression contributed to the phenotype of vMNTFG KO. Neither significant weakness, i.e., reduced muscle function, nor atrophy was evident in the MUSTFG KO mice, indicating the phenotypic features of vMNTFG KO to be attributable mainly to TFG deletion in motor neurons. However, further examinations revealed that mRNA levels of a denervation marker, AChRγ, were slightly upregulated in several muscles and that the increase was statistically significant in QF from the MUSTFG KO mice. The experiments using C2C12 myotube cells suggested that muscle TFG is necessary for agrin-induced AChR clustering. Given that agrin has been proven essential not only for NMJ formation but also for maintenance of adult NMJs^27–30^, decreased muscle TFG in vMNTFG KO and in aged mice may well be a factor exacerbating NMJ denervation in response to motor neuron degeneration or with aging. However, it is noteworthy that the upregulation of AChRγ was apparently more marked in muscles containing slow fibers (i.e., SOL, GAST, QF) rather than in fast muscles (i.e., TA, EDL) (Fig. 5a), a feature that was quite prominent when assessed in 4-month-old MUSTFG KO (Suppl Fig. S4g). This suggests that the agrin-induced AChR clustering defect is not sufficient to cause NMJ denervation in general and that additional factor(s), which appear to predominate in slow-twitch fibers, might also play a role. Mitochondrial dysfunction^37^, which was observed in TFG-depleted adipocytes (our unpublished observation), might be among these additional factors, but further studies are needed to fully elucidate the underlying mechanisms.

Intriguingly, muscle TFG was reduced in several muscles from aged mice. Considering that muscle TFG seems to be important in NMJ innervation, we speculate that lack of TFG in muscle triggers denervation and possibly muscle atrophy with aging in long-lived species such as humans, while not being detectable (at least histologically) in mice at least when assessed at 6–7 months of age. Further study is warranted to explore the possibility that muscle TFG reduction is involved in the development of sarcopenia or age-related muscle weakness.

To briefly summarize our findings, while TFG deletion in motor neurons results in marked dysfunction and atrophy of muscles, reduction of muscle TFG also appears to contribute to degeneration of NMJs (Fig. 6c). Both vMNTFG KO and MUSTFG KO, potential animal models of human neurodegenerative diseases including HMSN-P and NMJ degeneration with aging, hold promise as useful tools allowing further investigation of the pathogenesis of these disorders and the development of novel drug treatments.

## Supplementary Information

Supplementary Information.
