# Supplementary material for: Involvement of neuronal and muscular Trk-fused gene (TFG) defects in the development of neurodegenerative diseases
Source: Sci Rep. 2022 Feb 4;12:1966. doi: 10.1038/s41598-022-05884-7 (PMC8816932; doi:10.1038/s41598-022-05884-7)
Supplement: Supplementary file 1 — Supplementary Information. [file 41598_2022_5884_MOESM1_ESM.pdf]

Involvement of neuronal and muscular Trk-fused gene (TFG) defects in the development of neurodegenerative diseases

Takeshi Yamamotoya[1], Shun Hasei[1], Yasuyuki Akasaka[1], Yukino Ohata[1], Yusuke Nakatsu[1], Machi Kanna[1], Midori Fujishiro[2], Hideyuki Sakoda[3], Hiraku Ono[4], Akifumi Kushiyama[5], Hidemi Misawa[6], Tomoichiro Asano[1, *]

Supplementary Information

Supplementary Methods

Electron microscopy, Toluidine blue staining

Mice were anesthetized, perfused with saline and subsequently with fixative solution (2.5% glutaraldehyde, 2% paraformaldehyde in 0.1M phosphate buffer (pH 7.4)). Right sciatic nerves were excised, immersed in fixative solution overnight at 4°C and subsequently treated with 1.5% osmium tetroxide for 1.5 h at 4°C. The specimens were dehydrated in a graded ethanol series and then embedded in Epon 812. Ultrathin sections were obtained using a diamond knife and then stained with uranyl acetate and lead citrate, and observed by transmission electron microscopy using JEM-1400 (JEOL, Tokyo, Japan) operated at 80 kV. For toluidine blue staining, sections were heated at 70°C on a hot-plate, covered by 0.05% Toluidine Blue Solution (pH 7.0) (Fujifilm Wako, Osaka, Japan) for 2 min and then washed with water.

Quantification of OPN intensity

OPN intensities of lumbar spinal ChAT-positive motor neurons were quantified using Image J. Motor neurons were manually outlined and mean intensities were measured, and the values obtained were then subtracted average mean intensities of three backgrounds from the same section. As shown in Suppl Fig. S3d,e, we set the cut-off value at 12 A.U., taking into account the previous report[24] classifying ~20% of ChAT-positive neurons as OPN-high.

Supplementary Figure legends

Supplementary Figure S1. Related to Figure 1.

(a) Fluorescent immunostaining against OPN and TFG in lumbar spinal cord specimens from 6-month-old mice (scale bar: 100 μm). OPN-high, TFG-negative motor neurons in vMNTFG KO are indicated by arrowheads.

(b-f) Western blotting against TFG and actin was conducted using whole cell lysates from various tissues.

Supplementary Figure S2. Related to Figure 2.

(a) Body weights of 7-month-old TFG f/f mice (f/f) and vTFGKO mice (KO).

(b) Immunostaining of SOL against MyHC type 1 (scale bar: 100 μm, n = 5).

(c, d) mRNA levels of MyHC, Cox8b and atrogenes in GAST (c) and TA (d).

(*: $P < 0.05$, **: $P < 0.01$)

Supplementary Figure S3. Related to Figure 3.

(a) ChAT staining of lumbar spinal cord (left panel) (scale bar: 100 μm). The number of motor neurons per ventral horn (right panel). ChAT-positive motor neurons, with cell body diameters of at least 20

μm, were counted (6-month-old mice, n = 5-7).
(b, c) Toluidine blue staining (b) (scale bar: 40 μm) and electron microscopic images (c) (scale bar: 10 μm) of sciatic nerve.
(d) Histograms of OPN intensities in ChAT-positive motor neurons of lumbar spinal sections (6-month-old mice, n = 5). The cut-off point (12 A.U.) is indicated by the dotted lines.
(e) Percentages of OPN-high neurons among ChAT-positive motor neurons.

Supplementary Figure S4. Related to Figure 5.
(a) Treadmill test results of 3-month-old mice (n = 7-9). Distance (left panel) and time (right panel) that mice ran are shown.
(b) Latency to fall in hanging-wire test results of 3-month-old mice (n = 7-9).
(c, d) Body weight (c) and muscle mass (bilateral) adjusted by the body weight (d) (6- to 7-month-old mice, n = 10-11).
(e) IGF-1 concentrations in GAST homogenates (6-month-old mice, n = 5, adjusted by the protein concentration).
(f) Whole-mount staining of SOL from 6-month-old MUSTFG KO (left panel) mice (scale bar: 100 μm). Green: AChR stained by α-bungarotoxin. Magenta: Synaptophysin as a pre-synaptic marker. White arrowhead indicates completely denervated NMJ. Percentage of innervated neuromuscular junctions in SOL from MUSTFG KO (right panel) (6-month-old mice, n = 3). Both completely and partially innervated NMJs were defined as being innervated.
(g) Relative *AChRγ* mRNA levels in each muscle from 4-month-old TFG floxed (f/f) or MUSTFG KO (KO) mice (n = 6-8).

Supplementary Figure S5. Blot images for Fig. 4, 5, 6 and Suppl Fig. S1

Supplementary Table S1. qPCR primers used in this study

Supplementary Figure S1

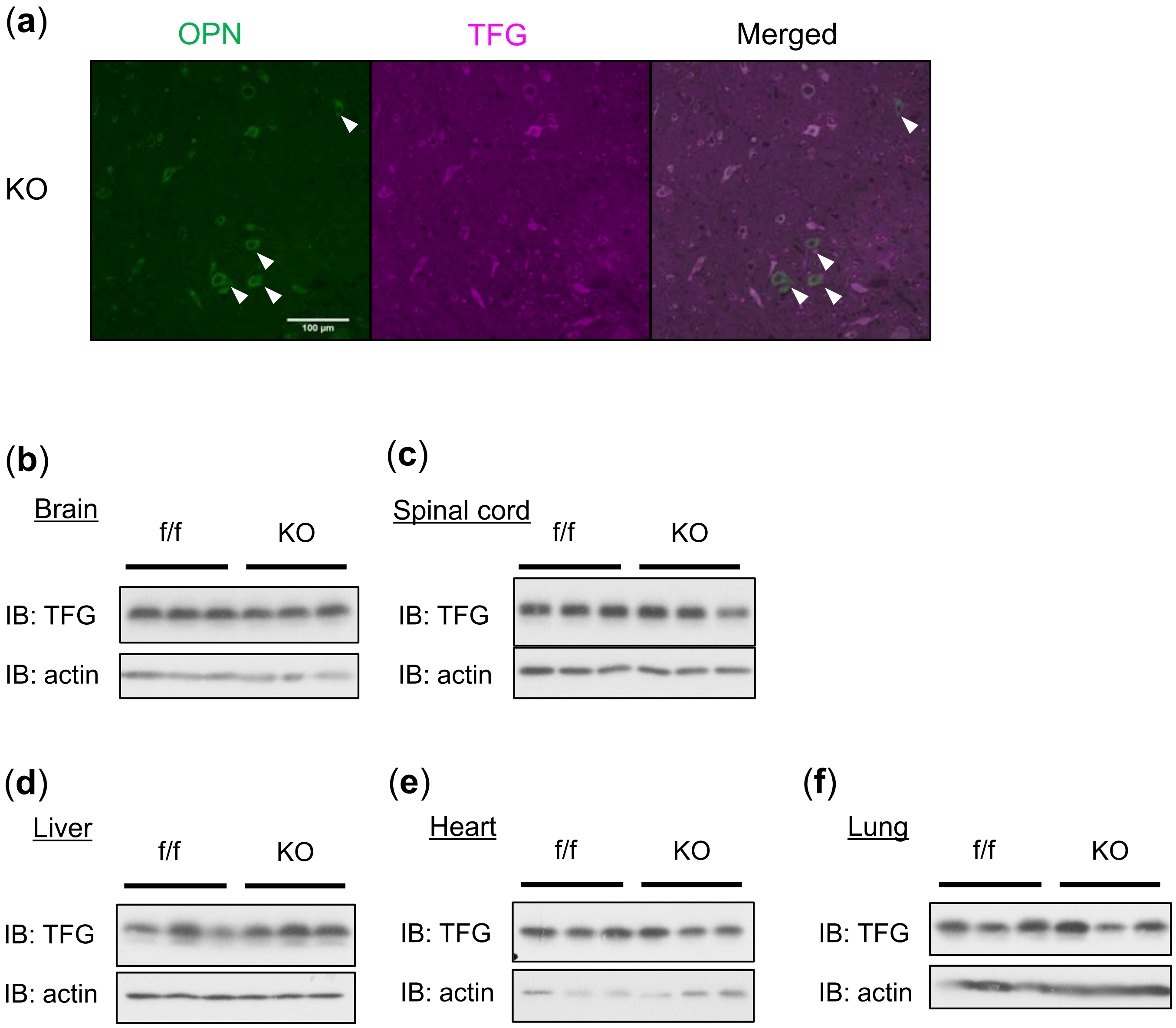

Supplementary Figure S2

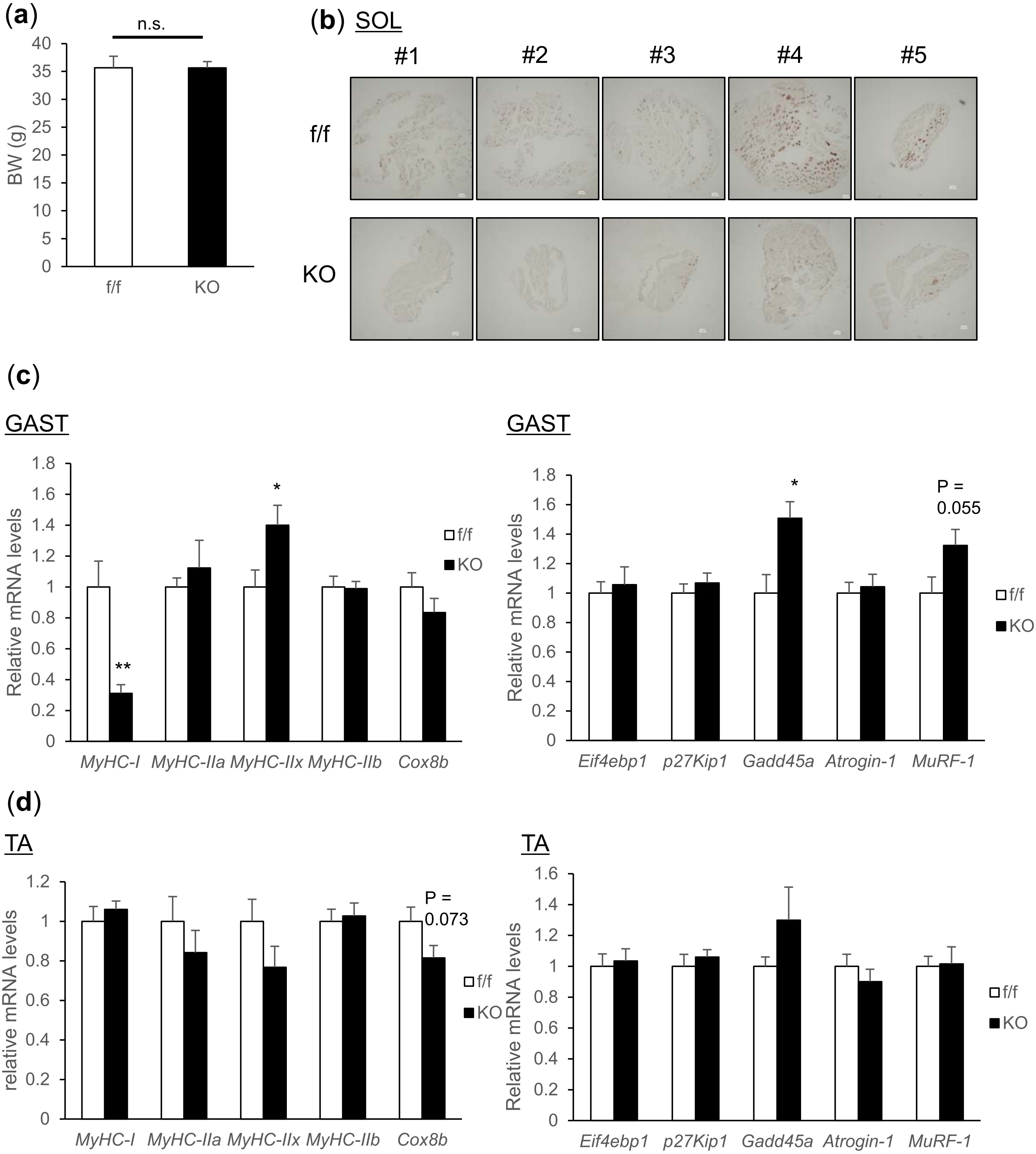

Supplementary Figure S3

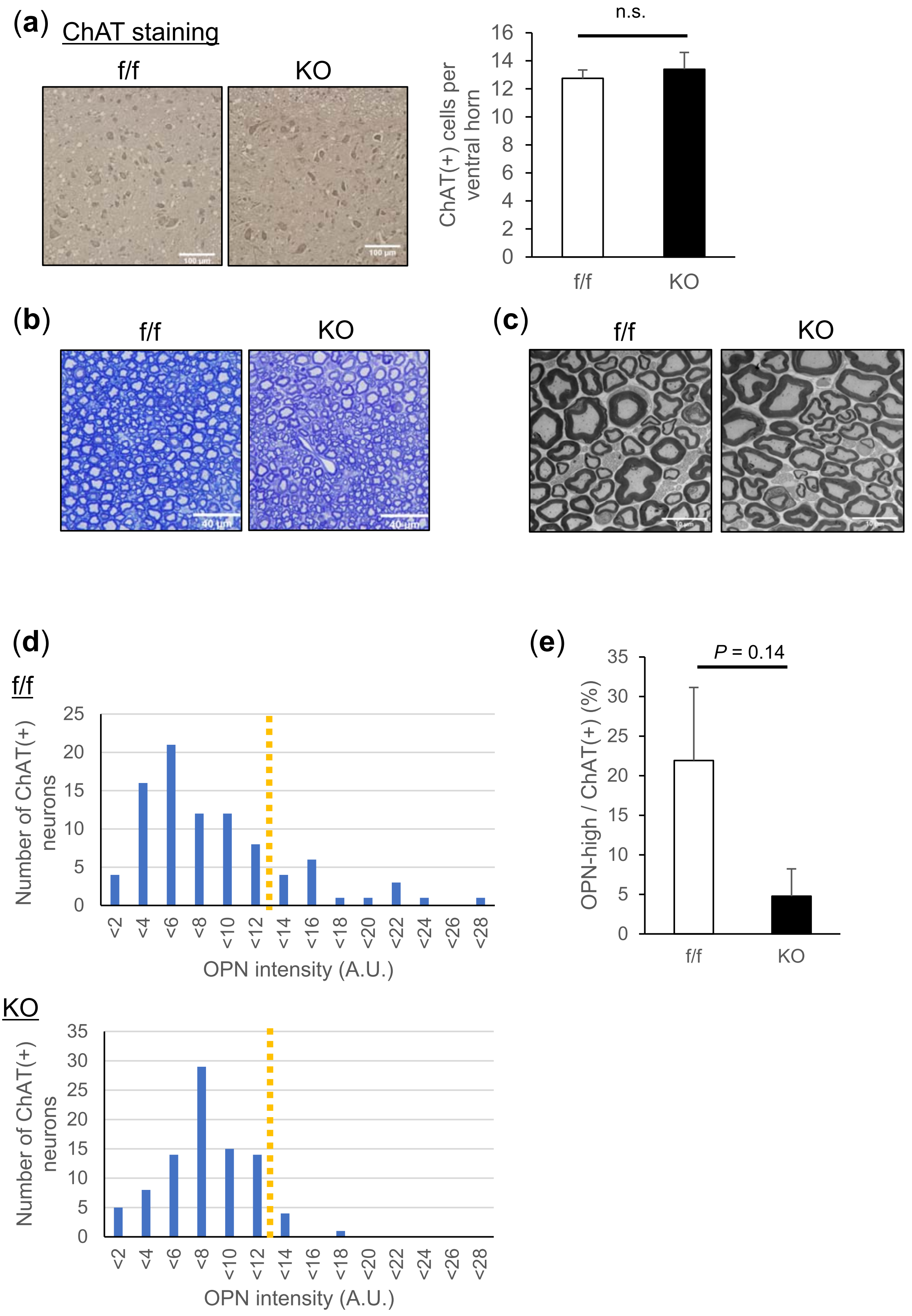

Supplementary Figure S4

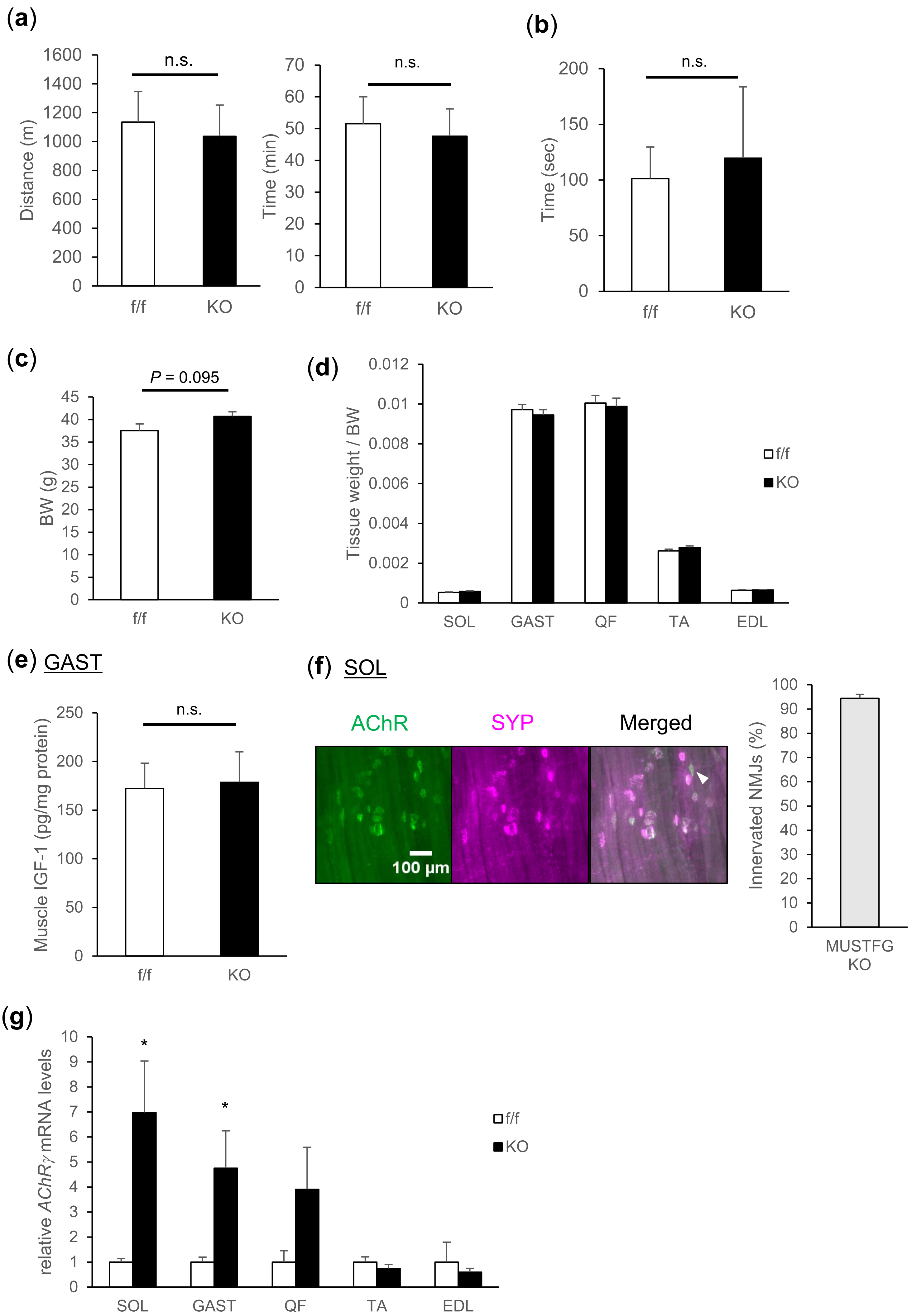

Supplementary Figure S5

Fig. 4

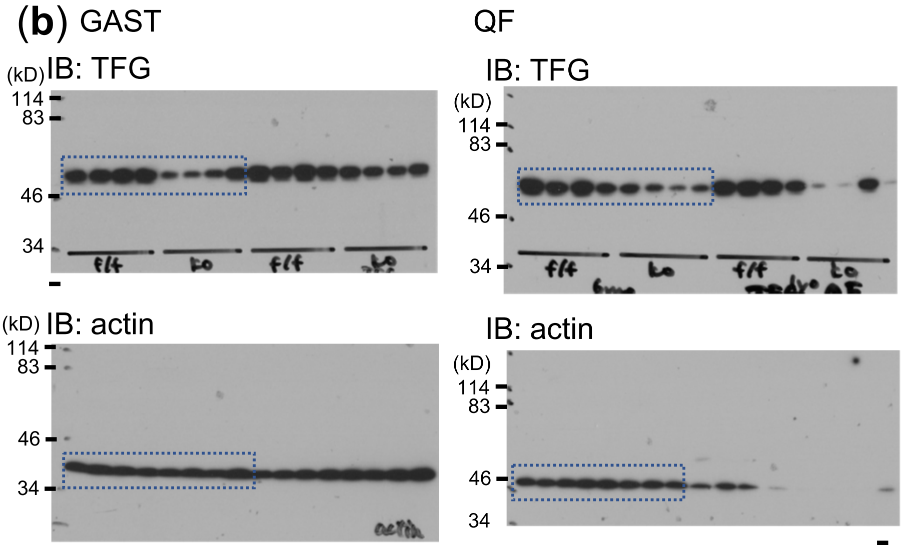

Fig. 5

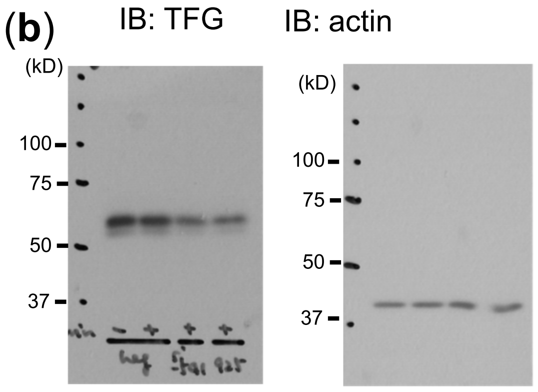

Fig. 6

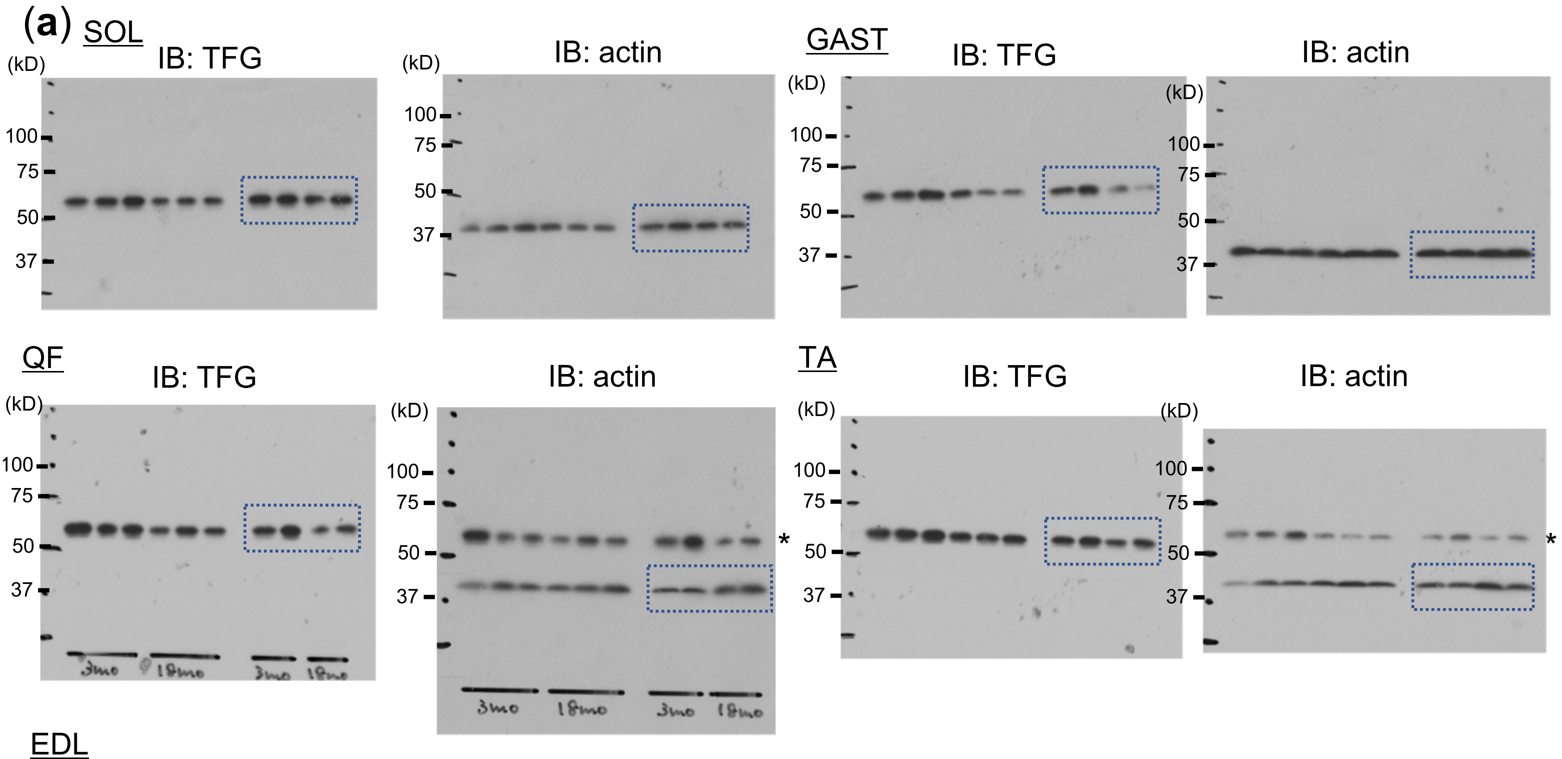

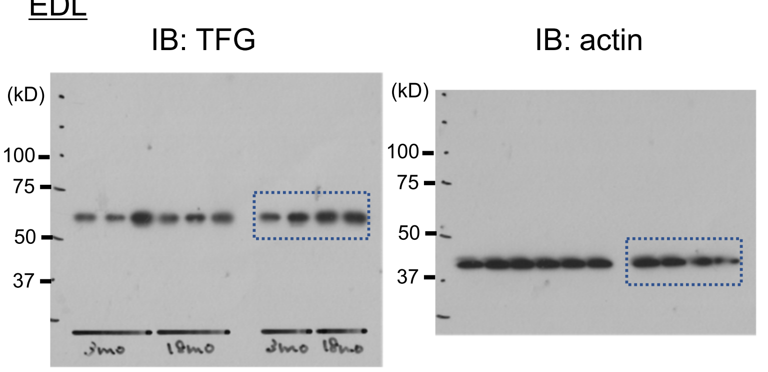

*: bands of TFG (, since these membranes were reblotted with actin antibodies after the TFG blotting)

Supplementary Figure S5

Fig. S1

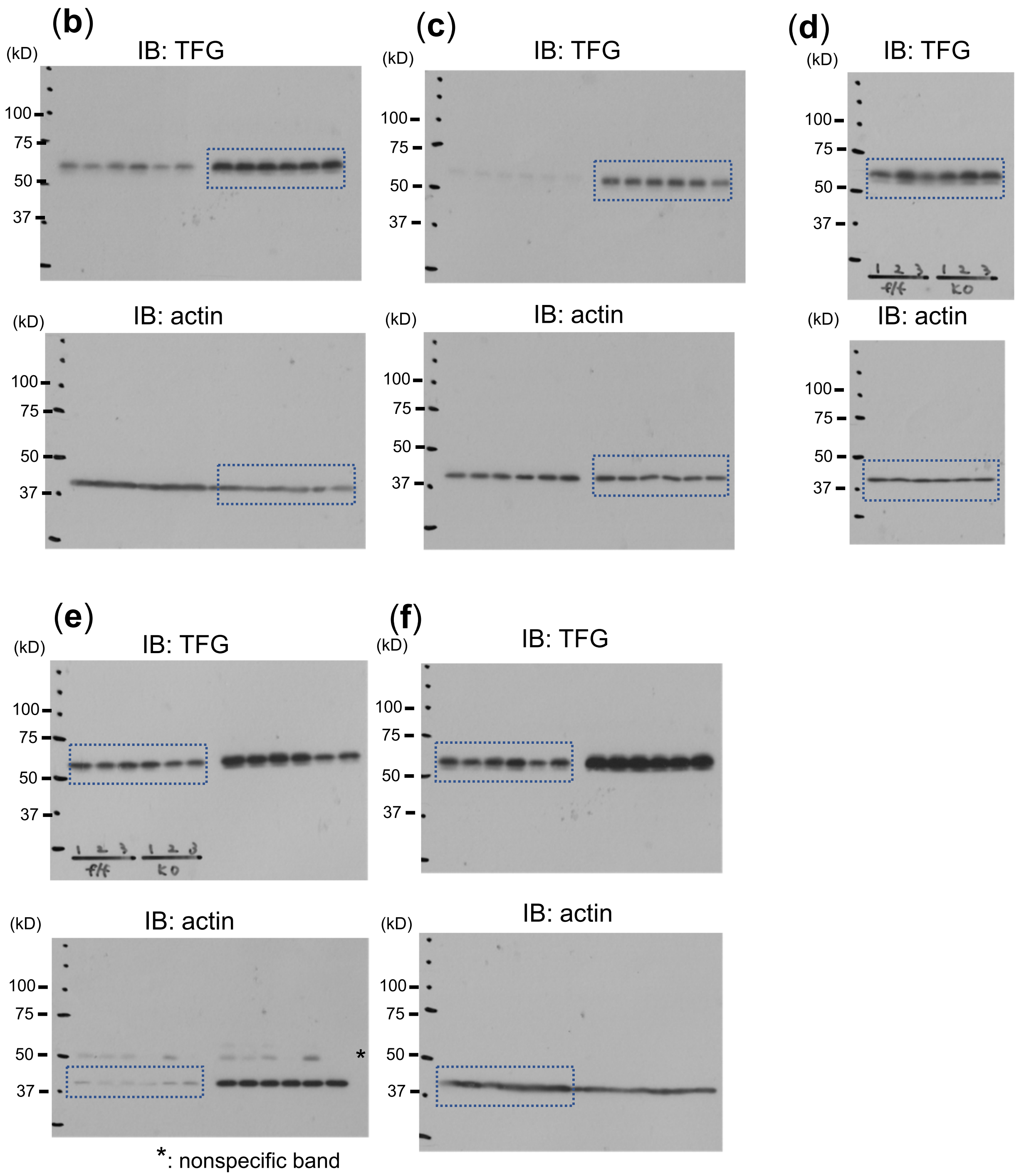

*: nonspecific band

Supplementary Table S1

	Forward	Reverse
Gapdh	TGATGGGTGTGAACCACGAG	GGGCCATCCACAGTCTTCTG
Tfg	CCTCGGCAAGCGGGTTTTT	AGCTTTCCTCTGAATACTCGCT
MyHC-I	AGATGAATGCCGAGCTCACT	CTCATCCAAACCAGCCATCT
MyHC-IIa	AATCGAGGCCCAGAATAGGC	TCTTCTTTCACGGTCAGGGT
MyHC-IIx	CAAGACCGAAGGCGGAACTA	TGACAGTGACGCAGAACAGG
MyHC-IIb	ACGCTTGCACACAGAGTCAG	TCACAGTCATGGCGAGCTG
Cox8b	AAGCCCATGTCTCTGCCAAG	CTTCATGCTGCGGAGCTCTT
Eif4ebp1	CTGATGGAGTGTCGGAACTCA	AGGTATGTGCTGGTGTTCACA
p27Kip1	AGAACTAACCCGGGACTTGG	CGGGGGCCTGTAGTAGAACT
Gadd45a	GAAAGTCGCTACATGGATCAGT	AAACTTCAGTGCAATTTGGTTC
Atrogin-1	CTGTGCTGGTGGGCAACATTAACA	CGTCACTCAGCCTCTGCATGAT
MuRF-1	ATGAAGTGATCATGGACCGGCA	TTGCACAAGGAGCAAGTAGGCA
AChRγ	GACCAACCTCATCTCCCTGA	GAGAGCCACCTCGAAGACAC
Igf1	TGCTCTTCAGTTCGTGTG	ACATCTCCAGTCTCCTCAG
Igf1r	AACACTGGCATCCAGGAGGA	GTCCAGGTCTCCCAATGCAG
